# Genomics of Smoldering Multiple Myeloma: Time for Clinical Translation of Findings?

**DOI:** 10.3390/cancers13133319

**Published:** 2021-07-01

**Authors:** Marta Lionetti, Matteo C. Da Vià, Francesco Albano, Antonino Neri, Niccolò Bolli, Pellegrino Musto

**Affiliations:** 1 Hematology Unit, Fondazione IRCCS Ca’ Granda Ospedale Maggiore Policlinico, 20122 Milan, Italy; marta.lionetti@unimi.it (M.L.); matteo.davia@unimi.it (M.C.D.V.); antonino.neri@unimi.it (A.N.); 2 Department of Oncology and Hemato-Oncology, University of Milan, 20122 Milan, Italy; 3 Department of Emergency and Organ Transplantation, “Aldo Moro” University School of Medicine, 70124 Bari, Italy; francesco.albano@uniba.it; 4 Unit of Hematology and Stem Cell Transplantation, AOUC Policlinico, 70124 Bari, Italy

**Keywords:** multiple myeloma, asymptomatic stages, genomic alterations, tumor evolution

## Abstract

**Simple Summary:**

In this review we summarized the most relevant biological features concerning smoldering multiple myeloma (SMM). We outlined the genetic architecture of the disease and how it is entering in the SMM risk stratification. In particular, we pointed out how the identification of a high-risk setting, meaning the population with the risk of faster progression to the symptomatic phase, is crucial and, despite huge improvements in recent years, still represents an unmet clinical need. Indeed, the correct identification of these patients will drive to an early therapeutical intervention. Moreover, we also discussed the role of the microenvironment, which is highly relevant in the symptomatic disease but still understudied in the SMM setting. Finally, we debated the state-of-the-art current available therapies and ongoing clinical trials, and envisioned possible strategies to introduce a biological-based stratification approach within the daily clinical practice.

**Abstract:**

Smoldering multiple myeloma (SMM) is an asymptomatic disorder of clonal bone marrow (BM) plasma cells (PCs) in between the premalignant condition known as monoclonal gammopathy of undetermined significance and overt multiple myeloma (MM). It is characterized by a deep biological heterogeneity that is reflected in a markedly variable progression risk among patients. Recently proposed risk stratification models mainly rely on indirect markers of disease burden and are unable to identify cases in whom clonal PCs have already undergone the “malignant switch” but major clonal expansion has not occurred yet. In the last years, the application of next-generation sequencing (NGS) techniques has led to profound advances in the understanding of the molecular bases of SMM progression, and in all likelihood, it will contribute to the needed improvement of SMM prognostication. In this Review, we describe the recent advances in characterizing the genomic landscape of SMM and intrinsic determinants of its progression, highlighting their implications in terms of understanding of tumor evolution and prognostication. We also review the main studies investigating the role of the microenvironment in this early disease stage. Finally, we mention the results of the first randomized clinical trials and discuss the potential clinical translability of the genomic insights.

## 1. Introduction

Smoldering multiple myeloma (SMM) is an asymptomatic disorder of clonal bone marrow (BM) plasma cells (PCs). SMM incidence is roughly 0.4–0.9 cases per 100,000 persons per year [1,2]. It represents an intermediate clinical stage between the premalignant condition known as monoclonal gammopathy of undetermined significance (MGUS), characterized by a lower clonal burden, and overt multiple myeloma (MM) [3]. Previously distinguished from overt MM strictly on the basis of clinical symptoms, SMM definition has recently been revised and currently provides that both of the following criteria are met: serum monoclonal protein (IgG or IgA) ≥30 g/L or urinary monoclonal protein ≥500 mg per 24 h and/or clonal bone marrow plasma cells (BMPCs) 10–60%; absence of myeloma defining events (MDEs) or amyloidosis (Table 1) [4].

The revision of diagnostic criteria made it possible to reclassify as MM a subset of asymptomatic patients previously considered SMM but having a ≥80% risk of progression within two years. Despite this update, SMM remains a clinically defined entity characterized by a deep biological heterogeneity that is reflected in a markedly variable progression risk among patients. An aggregate progression risk of about 10% per year for the first five years, 3% per year for the next five years, and 1% per year thereafter, in fact, is calculated by averaging the clinical course of two biologically distinct subsets of SMM patients: i) MGUS-like patients (roughly 30%), affected by what is likely a premalignant condition from a biological point of view, and characterized by a very low rate of progression, and ii) MM-like patients (approximately 70%) with biological malignancy who have not yet developed CRAB (hypercalcemia–renal failure–anemia–bone lesions) symptoms and/or other MDEs but are going to do so in the short to medium term. This framework clearly brings out the limits that characterize the arbitrarily determined clinical criteria reflecting tumor burden currently in use for the diagnosis of monoclonal gammopathies, and consequently the need to better define what distinguishes the patients in whom a malignant switch of clonal plasma cells has occurred from those who have only clonal benign/premalignant PCs.

## 2. Risk Stratification Models

**Figure 1:**
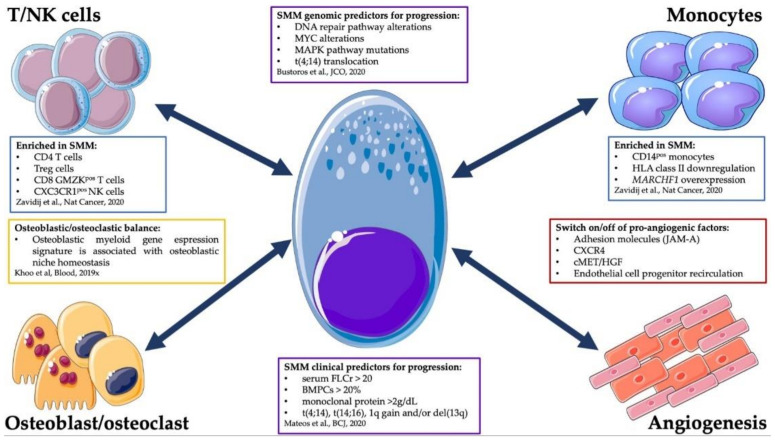
Interplay between multiple myeloma plasma cells and surrounding immune and non-immune microenvironment. This figure was created using Servier Medical Art templates, which are licensed under a Creative Commons Attribution 3.0 Unported License; https://smart.servier.com (accessed on 15 April 2021). Mateos et al. [6], Bustoros et al [8], Zavidij et al [9], Solimando et al [10], Khoo et al. [11].

For this purpose, risk stratification models aimed at predicting SMM progression are proposed (Table 2). The two main stratification models are the Mayo Clinic Criteria [5] and the International Myeloma Working Group (IMWG) risk stratification model [6], the former being a subset of the bigger IMWG multicenter initiative. Both are mainly based on indirect measurements of disease burden, while providing for the possibility of including cytogenetic abnormalities as additional prognostic factors contributing to determine the risk of disease progression. Serum M-protein >2 g/dL, ratio of involved to uninvolved serum free light chains (FLCr) >20, and >20% clonal PCs in the BM are independent predictors of shorter time to progression (TTP) in both analyzed patients’ cohorts, each representing one risk factor. Patients’ stratification based on the presence of 0 (low), 1 (intermediate), or ≥2 (high) risk factors identified three groups with significantly different median TTP. The risk of progression at two years in the low, intermediate, and high-risk group was, respectively, 6%, 18% and 44% in the IMWG study, and 10%, 26% and 47% in the Mayo Clinic cohort. In the former, the inclusion of high-risk cytogenetics [t(4;14), t(14;16), 1q gain and/or del(13q)] defined four groups of SMM patients with different progression risk at two years: 6% in the low (0 risk factors), 23% in the low-intermediate (1 risk factor), 46% in the intermediate (2 risk factors), and 63% in the high-risk (≥3 risk factors) groups (Figure 1). With the inclusion of presence versus absence of del(17p), and/or t(4;14) and/or hyperdiploidy in the Mayo Clinic multivariate model, BMPCs > 20%, FLCr >20, and high-risk cytogenetics were associated with higher risk of progression, and patients with none, one, and two or three risk factors showed significantly different progression rates (two-year risk of progression was 6%, 32% and 69%, respectively).

Models of this kind have proved useful, however, they have some limitations. Firstly, in terms of robustness, head-to-head comparisons of different risk models show considerable discordance in the overall risk stratification [12]. Furthermore, relying mainly on indirect markers of disease burden and not on intrinsic features that are true markers of biological disease characteristics, and as such determinants of its clinical behavior, they are unable to identify cases in whom clonal plasma cells have already undergone the “malignant switch” but major clonal expansion has not occurred yet [13].

Intrinsic biological features potentially able to more accurately define individual patients’ risk have been identified, i.e., recurrent translocations and copy number alterations (CNAs) detected by fluorescent in situ hybridization (FISH) [14,15,16] and gene expression profiles (GEP) [7,17]. Risk models using these alterations as markers of SMM progression, however, were not built by incorporating the 2014 IMWG revised SMM diagnostic criteria, and, furthermore, GEP-based ones suffer from poor reproducibility; therefore, their application is limited.

In all likelihood, the application of next-generation sequencing (NGS) techniques will contribute to the needed improvement of SMM prognostication, since NGS in recent years has led to profound advances in the understanding of the molecular bases of SMM progression.

## 3. Genomic Landscape and Intrinsic Determinants of SMM Progression

Several studies demonstrated that MGUS and SMM share the founding genetic alterations characterizing MM, namely recurrent translocations of oncogenes under the control of the IGH promoter and hyperdiploidy. However, some of these were observed at lower prevalence in asymptomatic disease stages as compared to MM, suggesting they may confer higher risk of progression [18,19,20,21,22]. Furthermore, although to a lesser degree than the fully malignant counterpart, the genomic landscape of MGUS and SMM is still complex and heterogeneous. Only with the recent application of NGS techniques, and in particular whole-genome sequencing (WGS), it was possible to have a more complete picture of the lesions involved in SMM progression [8,23,24,25], capturing also structural variants (SVs) and complex genomic aberrations involving non-coding sequences, which play a key role as disease drivers [26,27]. In particular, WGS, in addition to having uncovered an unprecedented catalog of MM driver events, has allowed estimating the timeline in which they are acquired during disease progression. Such a reconstruction of the natural history of MM evolution was made possible by novel computational approaches relying on cancer cell fraction and able to define the relative order of clonal somatic variants [28]. Specifically, in an attempt to characterize the early stages of myelomagenesis based on the chronological activity of each mutational signature, it has been estimated that the initial transformation of a germinal center B-cell usually occurs during the first 2nd to 3rd decades of life, thus preceding the clinical detection of MGUS, SMM or MM by decades [29].

### 3.1. Genomic Landscape

Two recently published NGS-based studies provide a comprehensive view of the genetic make-up of SMM [8,24]. Both studies highlighted that, overall, SMM has a similar genetic landscape to newly diagnosed MM. Bustoros et al. analyzed 214 and 48 SMM at diagnosis by whole-exome sequencing (WES) and deep target sequencing, respectively [8]. In five patients, WES analysis was repeated on a serial BM aspirate, sampled at least one year apart from the diagnostic one. Immunoglobulin heavy chain (IGH) translocations were identified in 36% of patients. CNAs were the most common genetic alterations (88%), with hyperdiploidy (HD) found in 55% of cases, and, at whole chromosome and arm-level, significant trisomies of odd-numbered chromosomes involved in HD, gain of 1q, del(13q) and del(16q). Significant focal deletions affected 1p22.1, 6q27, 14q24.3 and 14q32.31. Single nucleotide variants (SNVs) in genes significantly mutated in MM were present in 55% of patients: of these, 46% had alterations in MAPK, 10% [including del(17p)] in DNA repair, 22% in NFkB, 21% in protein processing and 6% in cell cycle pathways, respectively. Biallelic events involving *TP53*, *RB1*, *CDKN2C*, *ZNF292*, *DIS3* or *FAM46C* were seen in 6% of SMMs. The clonality levels at which genetic alterations were observed in the patients’ cohort suggested that CNAs are largely founder events, except for del(17p) and del(1p), which were mostly subclonal. On the contrary, mutations in the MAPK pathway were mainly later events that contributed to tumor progression. Boyle et al. reported on a targeted NGS-based cross-sectional study of 82 untreated SMM patients compared with a published data set of 223 MM patients analyzed by the same approach [24]. In nine patients, analysis of multiple sequential samples was provided [24]. The frequency of IGH translocations in SMM (35%) was identical to that seen in MM (37%). Adverse risk genetic lesions were less frequent in SMM compared with MM: among these, t(4;14), *MYC* rearrangements, del(1p), del(8p), del(14q), del(16q) and del(17p). At gene-level, fewer *FAM46C* and *NRAS* mutations were detected in SMM, with a trend towards fewer *KRAS* SNVs, suggesting that these alterations are MM defining events which, when present, identify cases in the process of transformation. At pathway-level, the MAPK and the NFkB pathways were significantly less frequently affected by mutations in SMM, with a trend observed also for DNA repair pathway. Biallelic events at tumor suppressor gene loci of relevance to MM at relapse were observed less frequently in SMM, suggesting second hits are a distinctive feature of the transition to MM.

### 3.2. Molecular Models of Progression

The unifying finding emerging from different studies based on NGS of paired MGUS/SMM-MM patients’ samples is the evidence of intraclonal heterogeneity and subclonality from the earliest MGUS/SMM stages, with most of the transformed subclonal populations involved in progression to MM already present at diagnosis [8,23,24,25,30,31]. There is no significant difference between the load of genomic alterations in progressive and non-progressive asymptomatic disease forms and between MGUS/SMM and overt MM. Rather, it is likely the pattern of mutations and genomic changes that may impact the risk of developing a clinically overt malignancy. A consensus exists on the notion that most of the genetic alterations characterizing overt MM have already occurred by the time of SMM diagnosis, and they constitute the substrate of the clonal evolution observed during progression, with subclonal cancer cell fractions changing over time. Against this background, the various studies (each conducted on a limited number of patients) differentially identified the preponderant contribution of an evident clonal evolution [8,24] or a substantial “clonal stability” (which provides that subtle changes in the existent subclonal structure and a degree of emergence and/or extinction of child subclonal branches underlie progression) [25], or both of these scenarios [23].

By analyzing the highest number (53) of sequential samples from nine SMM patients (of whom six underwent progression during follow-up time), Boyle et al. suggested that while HD, which is one of the major initiating events of MM, is stable and does not constitute a significant mechanism impacting TTP, segmental CN gains and losses are the key mechanisms underlying the transition to MM, with sub-clones carrying these abnormalities undergoing clonal advantage over time [24]. The monitoring of subclonal architecture in multiple sequential samples highlighted increasing diversity occurring over time and reflecting the ongoing acquisition of a more complex clonal structure, with branching evolution as the predominant pattern of progression. Importantly, changes in sub-clonal structure preceded clinically relevant events, and according to the Authors this could help in identifying SMM requiring treatment at a time of disease evolution in which the benefit–risk ratio is maximum [24].

The study by Bolli et al., on 11 SMM cases at diagnosis and paired tumor samples at the time of progression for 10/11, is the first based on WGS analysis [23]. Notably, all cases were classified as high-risk based on pre-2014 criteria and all evolved to symptomatic MM with a median TTP of eight months. The wealth of information provided by WGS highlighted a much richer catalog of genetic lesions (mutations, CN changes and rearrangements) compared to previous WES studies. At symptomatic MM stage, the Authors observed similar numbers of SNVs and indels, but importantly a relevant fraction of shared mutations significantly shifted their cancer cell fraction (CCF), and others were lost or acquired, suggesting the existence of a dynamic competition between subclones during disease progression. Analysis of paired samples showed two general patterns of evolution to symptomatic disease: a “spontaneous evolution model” where acquisition of new genetic lesion(s) conferred a proliferative advantage to a subclone at the expense of others; and a “static progression model”, in which all subclones were equally represented in both SMM and MM samples, without any significant change in their subclonal structure. This latter scenario may have been overestimated due to the shallower median coverage of WGS, which potentially may have missed additional subclones present at low frequencies in the cancer samples. Branching evolution characterized patients generally displaying a longer TTP, suggesting that the change of the biology of the tumor into a more aggressive phenotype required more time for the acquisition of additional genomic lesions. Conversely, the relatively shorter TTP of cases with no significant change in their genomic structure suggested the presence of overtly transformed cells with slow impact on clinical manifestations, and it solely reflected the time needed to accumulate a sufficient disease burden to become clinically symptomatic, possibly with the intervention of extrinsic factors.

In line with the hypothesis that the landscape of genomic alterations present in the asymptomatic stages determines which pattern of progression will be undertaken, a very recently published study took advantage of multi-parametric flow-cytometry sorting and low-input WGS approach to profile 18 MGUS and compare them with WGS of 14 SMM and 80 MM patients. Findings were that, irrespective of the clinical MGUS or SMM classification, progressive and stable myeloma precursor conditions emerged as two biologically distinct entities, characterized by a different spectrum of mutations, CNAs, complex rearrangements and mutational signatures [27].

### 3.3. Molecular Models of Risk Stratification

Beyond the limited concordance in terms of specific lesions identified as prognostic in the relative patients’ cohorts, the two main published cross-sectional studies suggested that NGS analysis was able to identify genomic predictors of progression whose addition to current clinical risk models could significantly improve SMM prognostication [8,24]. In particular, in a subset of 85 untreated patients in clinical trial setting before progression to MM, MYC aberrations (translocations or amplifications), MAPK pathway mutations, DNA repair pathway alterations, t(4;14), del(1p), del(8p), biallelic deletion events and APOBEC-associated mutations were associated with a shorter TTP [8]. Furthermore, in a multivariable model accounting for the Mayo 2018 clinical risk stratification [5], MYC aberrations, alterations in DNA repair and MAPK pathways were independent risk factors for progression, whose addition to current clinical risk models significantly improved prediction of progression [8] (Figure 1). Likewise, in 77 patients evaluable for progression, Boyle et al. identified *KRAS* mutations associated with shorter TTP [24], suggesting that the lack of association with prognosis that they have in MM highlights their important role in the transition from asymptomatic to symptomatic stages of the disease, mediated by impacting on cell-cycle progression and promoting a clonal sweep. Furthermore, *KRAS* mutations had an additive effect in determining higher progression risk with IMWG [5] and GEP4 [17] risk scores.

### 3.4. Mutational Signatures and Chromosomal Events

In the cohort by Bustoros et al., the majority of mutations were attributable to signatures related to cell aging. APOBEC and AID contributed almost equally, however only APOBEC signature was reported as significantly enriched in patients who progressed [8]. Findings consistent with the role of aberrant APOBEC activity later in disease progression also emerged from the study by Boyle et al., where the contribution of this mutational signature was higher in MM than in SMM, and a trend towards more rapid progression to MM was observed in cases with an APOBEC signature >5% [24]. Furthermore, in the SMM setting APOBEC mutations were not associated with MAF translocations. Overall, signatures related to cell aging (SBS1 and SBS5) were the major contributors to the mutational patterns identified in SMM of this series [24]. By examining the role of mutational signatures at the SMM disease phase in serial samples, mutational processes emerged as stable over time and not defining the transition to MM [24]. The same stability of mutational processes was reported by Bolli et al. [23], who highlighted how all processes that shape the MM genome are already operative at the asymptomatic stages, as also indicated by the mutational signatures extracted by nonnegative matrix factorization (NNMF) and model selection approach. Indeed, SBS1 and SBS5 [32] and APOBEC signatures (SBS2 and SBS13) accounted for 23% and 13% of all substitutions, respectively, and two additional signatures were found, i.e., the noncanonical AID (ncAID) signature (#1, 28%) and one compatible with signature #8 (28%). When applied independently on each cluster of mutations, NNMF highlighted how the ncAID signature was particularly enriched in clonal events, while APOBEC and signature #8 accounted for most late substitutions. Based on this, a novel pathogenetic model for MM was hypothesized, where aberrant AID activity contributes to tumor initiation, and provides a fertile ground where other late processes (i.e., APOBEC and signature #8) act and shape the final genomic landscape of overt MM [23]. This is in line with the finding that APOBEC mutational process is particularly active in aggressive MM [33,34,35].

As regards chromosomal events, WGS analysis of 67 samples (11 of which at SMM stage) serially collected from 30 MM patients showed that major drivers involved in the early stages of myelomagenesis are complex structural events, such as chromothripsis and cycles of templated insertions, and hyperdiploidy. Positively selected point mutations, whole-genome duplication and chromoplexy, conversely, are potential new mechanisms of subclonal selection and treatment resistance, as suggested by their general acquisition during progression and/or relapse [26].

## 4. Cell-Extrinsic Determinants of SMM Progression

The immune-microenvironment (IME) and the non-IME represent one of the main actors in MM and in its progression from the asymptomatic to the symptomatic stages [9] (Figure 1). In particular, the MM PCs are dipped in a permissive ecosystem able to protect the tumor from immune surveillance, allowing its growth and escape from apoptosis. Of great interest, in the last years the bone marrow niche has emerged as a nurturing place, shaped by tumoral cells (solid and hematological ones) to their favor to reside, protected by a favorable microenvironment [36,37]. Indeed, tumor surveillance by the IME is likely to be effective at killing tumor cells in their initial stages, in what could be called the elimination phase. However, some cancers may alter the immune response (immune editing) reaching an equilibrium with the IME and, ultimately, escape [37]. As for solid malignancies, immune editing properties of MM have been clearly shown to foster disease progression in vivo using mouse models. The Vk*MYC model in particular represents a key animal model, where MM develops spontaneously [38]. Here, through a cross with a CD226-mutated mouse, Guillerey et al. showed the pivotal role of CD226 expression in the early phases of MM development [39], since CD226 downregulation promoted immune-evasion from NK and T cell surveillance and disease progression [39]. Interestingly, Carbone et al. demonstrated, using primary patient samples, the importance of the NK immune surveillance mediated by major-histocompatibility-complex class I receptors in MM cells and by the NKG2D activating receptor on NK cells. The authors speculated that this mechanism is particularly active in the early intra-medullary phases of the disease, as loss of NK immune surveillance was associated with disease progression and dissemination [40]. Interestingly, recent advances in the genotyping of MM precursor phases have timed the presence of a primordial clonal MM ancestor already to the third decade of life [29]. Arguably, the elimination stage could represent the micro-environmental failsafe mechanism controlling the cell-intrinsic errors leading to PC transformation already early in life and aging of the immune system could be another mechanism favoring the tolerance of small clones of bone marrow clonal PCs in the absence of an overt disease.

Indeed, the equilibrium phase represents the subsequent level of the disease-microenvironment adaptation. From an experimental point of view, the definition of this stage is very challenging, but in the continuous spectrum of PC dyscrasias we could assume that this microenvironmental stage parallels the MGUS or SMM phases. Using humanized immuno-deficient mouse models, Das et al. experimentally recapitulated the different growth patterns of pre-symptomatic and symptomatic stages of PC dyscrasias [41]. Xenografts of PCs derived from MGUS, SMM, symptomatic MM and plasma cell leukemia (PCL) showed different behaviors in this model, where immune surveillance was absent. In particular, the pre-clinical MGUS and SMM conditions grew only locally, but progressively to a larger extent than in their primary human host, where a cell-extrinsic mechanism linked to the IME was the likely restraint against disease progression [41]. On the other hand, MM and PCL samples showed an aggressive growth pattern, with the former invading the contralateral tibia and the latter showing an extramedullary invasion of the spleen, suggesting higher aggressiveness that was cell-intrinsically determined [41]. Once again supporting an equilibrium phase, Bailur et al. showed an enrichment of TCF1 high-expressor cells in MGUS patients [42]. These cells are specific memory T cells maintaining stem-like features [43]. During the asymptomatic phases, this enrichment could represent the reservoir of cells able to maintain the “equilibrium” whose loss could drive to the progression to an overt disease [42]. 

On another hand, the IME could also contribute actively to the disruption of this equilibrium phase. This apparent paradox was demonstrated by Calcinotto and colleagues, who showed that an increased proliferation of Prevotella heparinolytica within the gut microbiome of Vk*MYC mice was associated with a higher recruitment of Th17 T cells in the gut and in the BM. Th17 cells secreted high levels of interleukin-17 (IL-17), activating the STAT3 pathway in PCs and recruiting IL-6 producing eosinophils, thus promoting PC proliferation and SMM progression [44]. The composition of the IME has recently been dissected at the single cell level with functional implications by Zavidij et al. [9]. The authors demonstrated early modifications of tumor IME already at the MGUS stages. Here, an enrichment of NK, T and CD16+ cells was significant when compared to the BM of healthy donors. As already mentioned, in this study the role of NK cells seemed pivotal in early phases. Indeed, when investigating differences between MGUS and normal BM, the authors observed different rates of BM infiltration by NK cells with different phenotypes in MGUS. In cases with a high recruitment, NK cells expressed high levels of CXCR4, suggesting a more immature phenotype. On the other hand, when the infiltrate was less abundant, the CX3CR1 positive subset was more represented, indicating a more mature phenotype [9]. Concerning the T cell compartment, Zavidij et al. described different signatures, showing an enrichment of IFNα-producing CD4 cells and T regulatory cells in the more advanced stages. Moreover, within the cytotoxic T-cell compartment, the authors reported a depletion of CD8 memory T cells and higher levels of granzyme K effector T cells [9]. These differences in NK and T cell composition could imply immune editing by the tumor, impacting the equilibrium phase. Finally, the myeloid compartment also plays an important role in the creation of a permissive microenvironment. Indeed, in comparison with healthy donors, the IME of MGUS, SMM and MM samples were enriched for CD14+ monocytes that have internalized MHC class II antigen, likely through overexpression of the MARCHF1 gene, a E3 ubiquitin ligase regulating expression through internalization of these surface proteins. The downregulation of class II MHC receptors in CD14 monocytes has already been described in other cancers and is correlated with an acquisition of an immunosuppressive potential by these cells [9]. Altogether, this paper is to date the most insightful view of the IME of primary samples in MGUS, SMM and MM stage, and represents a great example of how the interaction between the tumor and the IME could shape a “friendly” ecosystem, fostering MM progression through asymptomatic stages to overt disease.

In addition, the non-IME plays a pivotal role in PC survival and proliferation within the BM niche. In particular, the neo-angiogenetic process seems to be involved in the progression phases of the disease. Indeed, within the BM niche several cell types can secrete pro-angiogenetic cytokines, including the neoplastic PC itself [10,45]. While in the pre-symptomatic phases of the disease angiogenesis is “switched off” or slowed down [46], it is highly activated during the progression to overt MM and in advanced MM [10,46,47,48]. At the moment, we do not know the mechanisms underlying this switch. Their understanding will contribute to more opportunities to predict or even control MM progression. The osteoblastic niche represents another key player in MM progression. Similar to their role in promoting quiescence in metastatic solid tumor cells, the osteoblastic niche has been recently shown to have a role in BM homeostasis maintenance in MM as well [49]. Indeed, one of the hallmarks of symptomatic MM is the development of bone lesions and an ancillary backbone of MM therapy is the anti-osteoclastic one, which has also shown a survival benefit in some settings [50]. We could argue that the pre-symptomatic stages are associated with a dormancy of the osteoclastic niche, whose reactivation could trigger MM progression to a symptomatic phase [11]. Indeed, in a mouse model the Croucher group recently showed that co-culture of MM and osteoblastic cells can induce in the former a state of dormancy characterized by a specific myeloid gene signature. The authors further translated this peculiar transcriptomic signature on primary samples from healthy donors, MGUS and symptomatic MMs [11]. Interestingly, this gene signature was highly expressed in the normal BMs and the pre-clinical phases and downregulated in the overt disease. Moreover, subsetting the analysis only on the symptomatic phases, patients with a higher expression of this gene pattern showed a survival benefit [11]. 

Taken together, these data show the complexity of the MM immune and non-immune microenvironment. In particular, the interconnection and the interactions between the PCs and the BM niche are fundamental to create a homeostasis between the tumor and the normal counterpart, escaping the elimination phase of the immune surveillance. The break of the subsequent equilibrium phase is then required to definitely escape immune surveillance and drive the progression from asymptomatic stages to overt MM. This view is also indirectly corroborated by the strong effect that immune-modulatory drugs, monoclonal antibodies, T-cell engager antibodies, and CAR-T cells have on the disease growth, and by the accepted notion that the simultaneous targeting of both tumor and microenvironment represents the better strategy to improve disease control.

## 5. Clinical Perspectives

In 2013, the Spanish group published the preliminary data of the first randomized trial on high-risk SMM, randomizing patients between a lenalidomide/dexamethasone treatment and observation. As well as the first results [51], also the long follow-up analysis [52] highlighted an improvement in the time to progression in patients who underwent treatment. Indeed, after 75 months of follow-up, the Lenalidomide group did not reach the median TTP, which, on the other hand, was of 23 months in the observation group, translating into a survival benefit [52]. Of great importance, the frequency of secondary tumors was higher in the Lenalidomide group but, taking into account the cumulative risk of cancer development, no statistically significant differences were noted [52]. Similar improvements in PFS, but not in OS, were reported by an American study where intermediate or high-risk SMM patients were randomized to lenalidomide versus observation [53]. Nevertheless, despite the promising results of these randomized clinical trials, clinical practice has not changed yet, as several still unresolved aspects impose caution in the interpretation of the data. Firstly, a more precise patient risk stratification which also considers the disease’s biological characteristics still represents an unmet clinical need. Furthermore, some open questions remain, to which ongoing clinical trials [54] will need to give a solid and definitive answer. Among others, what is the outcome we need to reach? Is it the delay of progression, or we could aim towards MM cure with an early intervention? To achieve this goal, do we have to intensify the treatment already at this stage? Furthermore, the start of a therapy in a patient with a more remote risk of developing non-organ damage requires a thorough assessment of the real risk–benefit ratio, including parameters such as the quality of life under treatment and short- and long-term side effects. In conclusion, despite the promising preliminary results of interventional trials and huge improvements in patients’ risk stratification, the therapeutical approach to SMM is still at its infancy, and biological milestones able to drive its wider application are still needed. This will allow, in the near future, to significantly prolong MM patients survival, preserving a good quality of life.

## 6. Conclusions

As extensively described above, in recent years the studies on the genomics of SMM have tried to identify the genomic correlates of the heterogeneity of the disease observed at the clinical level, undoubtedly providing important insights in this regard. However, these results are unlikely to have an immediate clinical translation. This is partly due to the fact that the mechanisms underlying the evolution to MM inferred by the reconstruction of clonal models of disease progression (i.e., changes in clonal substructure and genomic patterns of evolution) are hardly investigable in routine clinical practice [8,24,27,55]. Furthermore, at the level of specific mutations, translocations and copy number alterations, univocal driver lesions predictive of the transition to overt MM have not yet been defined [8,24]. Nevertheless, the study of SMM genomics, overall, is still in its infancy, and advances in this regard are conceivable by continuous research in this field. While WGS studies hold the promise of offering the best prognostication of SMM and MM, since they can highlight the presence of complex rearrangements and mutational signatures, their application to routine clinical practice is limited by cost and complexity of the downstream analysis. On the contrary, small targeted panels may hold enough value to allow personalized clinical decision-making in plasma cell dyscrasias. In the perspective of achieving this ultimate goal, it is worth underlying the promising prognostic impact that genomic analysis has demonstrated to have in the context of streamlined NGS approaches that can hopefully be integrated into the routine diagnostic framework of patients [56]. The feasibility of genomic analysis in routine clinical practice should be maximized by the employment of less invasive sampling techniques than BM biopsy, such as liquid biopsy. Recently published data indicate that even in the presence of a moderate tumor burden in the BM, sequencing of circulating cell-free DNA on the liquid biopsy is practicable and informative when performed at high depth of coverage, while it might not be sufficiently sensitive to detect tumor-specific aberrations by means of genome-wide, shallow sequencing [57].

## Figures and Tables

**Table 1:** Revised International Myeloma Working Group criteria for the diagnosis of SMM (both criteria should be met).

Feature	Required Extent
(1) Serum monoclonal protein (IgG or IgA)	≥30 g/L
or	
urinary monoclonal protein	≥500 mg per 24 h
and/or	
clonal bone marrow plasma cells	10–60%
(2) myeloma defining events (MDEs) ^1^ or amyloidosis	absent

^1^ MDEs are defined as: evidence of end-organ damage attributable to hypercalcemia, renal insufficiency, anemia, bone lesions (CRAB); clonal bone marrow plasma cell percentage ≥60%; involved/uninvolved serum free light chain ratio ≥100; >1 focal lesion on magnetic resonance imaging (SLiM).

**Table 2:** Prognostic models in SMM including biologic parameters (GEP, cytogenetics, genomics).

Model	Risk Factors	Risk Group	Two-Year PD Rate (%)
SWOG 2014 [7]	MC > 3 g/dl, sFLC > 25 mg/dl, GEP-70 > 0.26	Low (0 factors)	3.4
		Intermediate (1 factor)	21.9
		High (> 2 factors)	66.7
IMWG 2020 [6]	MC > 2 g/dL, sFLCr > 20, BMPC > 20% + high risk cytogenetics: [t(4;14), t(14;16), +1q, del(13q)]	Low (0 factors)	6
		Low-intermediate (1 factor)	22
		Intermediate (2 factors)	45.5
		High (3–4 factors)	63.1
Dana Farber 2020 [8]	DNA repair pathway gene alterations [mutations in *TP53* and *ATM*, del(17p)], MAPK pathway gene mutations (*KRAS*, *NRAS*), *MYC* aberrations (translocations or copy-number variations)	0 factors	14.4
		At least 1 factor	86.4

Abbreviations: BMPCs: bone marrow plasma cells; GEP-70: UAMS 70-gene expression profile signature; IMWG: International Myeloma Working Group; MC: monoclonal component; PD: progressive disease; sFLCr: serum free light chains ratio; SMM: smoldering multiple myeloma; SWOG: Southwest Oncology Group.
